# G.A.M.E.: GPU-accelerated mixture elucidator

**DOI:** 10.1186/s13321-017-0238-7

**Published:** 2017-09-15

**Authors:** Alioune Schurz, Bo-Han Su, Yi-Shu Tu, Tony Tsung-Yu Lu, Olivia A. Lin, Yufeng J. Tseng

**Affiliations:** 1 Graduate Institute of Biomedical Electronics and Bioinformatics, National Taiwan University, No. 1 Sec. 4, Roosevelt Road, Taipei, 106 Taiwan; 2 Department of Computer Science and Information Engineering, National Taiwan University, No. 1 Sec. 4, Roosevelt Road, Taipei, 106 Taiwan; 3 Drug Research Center, National Taiwan University College of Medicine, No. 1 Sec. 1, Jen Ai Rord, Taipei, 106 Taiwan

**Keywords:** Natural product, Mass spectrum, Structure elucidator, GPU acceleration

## Abstract

GPU acceleration is useful in solving complex chemical information problems. Identifying unknown structures from the mass spectra of natural product mixtures has been a desirable yet unresolved issue in metabolomics. However, this elucidation process has been hampered by complex experimental data and the inability of instruments to completely separate different compounds. Fortunately, with current high-resolution mass spectrometry, one feasible strategy is to define this problem as extending a scaffold database with sidechains of different probabilities to match the high-resolution mass obtained from a high-resolution mass spectrum. By introducing a dynamic programming (DP) algorithm, it is possible to solve this NP-complete problem in pseudo-polynomial time. However, the running time of the DP algorithm grows by orders of magnitude as the number of mass decimal digits increases, thus limiting the boost in structural prediction capabilities. By harnessing the heavily parallel architecture of modern GPUs, we designed a “compute unified device architecture” (CUDA)-based GPU-accelerated mixture elucidator (G.A.M.E.) that considerably improves the performance of the DP, allowing up to five decimal digits for input mass data. As exemplified by four testing datasets with verified constitutions from natural products, G.A.M.E. allows for efficient and automatic structural elucidation of unknown mixtures for practical procedures.

**Electronic supplementary material:**

The online version of this article (doi:10.1186/s13321-017-0238-7) contains supplementary material, which is available to authorized users.

**Graphical abstract:**
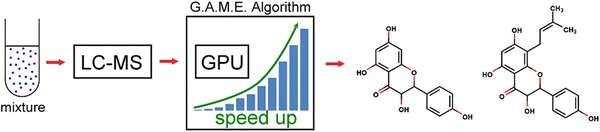
.

## Background

Mass spectrometry (MS) is one of the most widely used analytical methods for identifying the components of unknown mixtures or natural products. Elucidation of chemical structures, especially from natural products, is important to identify potential drug candidates with fewer adverse effects and structural novelty for drug discovery [1]. Mass spectra indicate the mass-to-charge ratio of each component, and the amplitude of each peak roughly represents the relative abundance of the molecule. However, additional techniques such as nuclear magnetic resonance (NMR) [2], a time-consuming and complex procedure, are needed to fully identify each component in the mixture. Furthermore, MS itself cannot elucidate the structure of a partially or completely unknown compound [3–5]. Therefore, successful structure elucidation of unknown compounds depends on the development of advanced computational tools for analyzing mass spectral data [6].

Several NMR-based expert systems have been developed to facilitate automated structure elucidation [7–15]. This type of computer-aided structure elucidation (CASE) approach suffers from the complex and time-consuming nature of NMR experiments, which usually involve multiple runs of different 2D NMR [16]. Moreover, the required NMR protocols may differ for different compounds, especially those with few hydrogen atoms or a diverse array of heteroatoms [14]. Additionally, the limited sensitivity of NMR relative to vibrational spectroscopy and mass spectroscopy further limits the development of high-throughput automated CASE expert systems [17, 18]. Recently, Harn et al. [19] have developed a new CASE method (NP-StructurePredictor) that can efficiently and accurately predict individual components in a mixture (The detailed method of NP-StructurePredictor is included in the Additional file 1); this approach is based on a model generated by referencing a collection of 226,949 natural products. NP-StructurePredictor divides each compound into a major scaffold and its sidechains. The relationship between each scaffold is initially constructed, and the probabilities of each sidechain on different possible scaffolds are evaluated. The model then takes the input seed scaffolds provided by users to match scaffolds in the model and automatically generates a list of most probable matching compounds sorted on the basis of evaluated probabilities for a given mass peak. The list is populated by combining possible sidechains at each position of the scaffold and evaluating the probability of the resulting compounds by using the natural products database (NPDB) probability data. Only the resulting compounds whose weight corresponds to the mass peak are retained. Experiments involving real test cases have shown that NP-StructurePredictor can correctly predict most of the compounds in mixtures, but it does so in a computationally inefficient manner.

The computational problem defined in the NP-StructurePredictor system has been formally proven to be NP-complete; it constitutes a chemical substituent core combinatorial problem (CSCCP) [20]. Because of the NP-completeness of CSCCPs, the branch and bound strategy used in NP-StructurePredictor lead to long running time for complex scaffolds. To resolve this issue, Su has developed a dynamic programming (DP) algorithm [20] to increase computational efficiency (the detailed method of DP algorithm is included in the Additional file 1). Although the DP algorithm allowed optimization of the NP-complete problem into pseudo-polynomial time, the search for optimal solutions can still be completed in exponential time complexity in the worst-case scenario. Each additional decimal digit of mass in the DP algorithm increases the running time tenfold. For example, the required computational time in the challenging case of *C. chinensis* would exceed 1 month if the number of decimal digits was set to 5. For high-resolution LC–MS experiments, the unreasonable execution time required to elucidate unknown chemical structures based on the DP strategy is still not functional in many cases.

To overcome the computational bottleneck of automatically identifying chemical components of mixtures with the DP algorithm, hardware acceleration via graphical processing units (GPUs) can be directly used to substantially improve the time performance. Indeed, the use of GPUs has recently attracted broad attention in the field of computer science. GPUs are not merely specialized video rendering devices to assist in accelerating the visualization of 3D graphics; they can also be used as a programming interface to support high-performance parallel computing [21]. The traditional CPU-based algorithm is no longer an effective way to solve extremely computationally demanding tasks [22]. A modern GPU contains thousands of efficient threads that can simultaneously perform multitasking optimization. Consequently, GPU methods are faster than conventional CPU methods and can provide an increase of one or two orders of magnitude [23]. Most importantly, the CUDA programming toolkit released by NVIDIA facilitates the development of software parallelism via GPUs. CUDA is an extension of the standard C/C++-like programming language, allowing researchers to implement and explore the parallel computing ability of GPUs. An increasing number of applications for GPU computing have been developed in different fields of cheminformatics, including free energy calculations [24], molecular docking [25, 26], molecular dynamics simulations [27, 28], high-throughput screening [29], similarity searching [30], and classification [31]. Therefore, the use of massively GPU parallel architecture is a feasible way to minimize the effects of NP-completeness complexity in CSCCP.

In this study, we present a GPU-accelerated algorithm, the GPU-accelerated mixture elucidator (G.A.M.E.), that can efficiently promote performance improvements of DP algorithms used to resolve chemical structures in the mass spectra of an unknown mixture. G.A.M.E. is a novel GPU-accelerated method implemented by the CUDA toolkit on NVIDIA cards that uses the heavily parallel architecture of modern GPUs. This method significantly decreases the computational time when five decimal digits of mass are required, thus allowing for efficient and automatic structure elucidation in practical cases. All datasets and source code of G.A.M.E. are available on GitHub at https://github.com/CMDM-Lab/GAME.

## Methods

The system architecture of G.A.M.E. is identical to the DP algorithm in previous study other than the GPU parallel programming in G.A.M.E. The system utilized molecular weights calculated from a list of m/z values obtained through LC–MS experiments as input information to predict chemical structures matching the input molecular weights in a mixture. Furthermore, possible scaffold structures of the mixture can be provided to the system to accelerate the prediction processes. When possible scaffolds cannot be provided, G.A.M.E. is able to apply all scaffolds collected in our database to search for suitable candidates. After such prediction by the GPU searching algorithm, a list of possible candidates in mixtures and their relative rankings will be provided. In the previous studies, four Chinese herb mixtures with verified structures were used to validate the accuracy of the NP-StructurePredictor system. Since G.A.M.E. is a new GPU programming version of the previous system with the same architecture, we will only focus on the improved time performance of the system in this study.

### Datasets

The G.A.M.E. algorithm searches for possible compounds matching the input molecular weights by expanding a seed scaffold with suitable sidechains. For comparing the feasibility and processing time between G.A.M.E. and its original CPU version, a natural product database (NPDB) was built from curating all of the known structures from the Dictionary of Natural Products (DNP) [32], the “ZINC natural products” subset of ZINC [33], and the Traditional Chinese Medicine Database [34]. The scaffolds are the remaining core structures after all terminal side chains have been deleted. Then, a total of 83,242 scaffold files were generated from the collected 243,130 natural products in the NPDB. The side chains excluded from the natural products were separated curated as our side chain database. Furthermore, we also analyzed possible sets of atoms (positions) on each scaffold structure that can be linked by the side chains, and evaluated linking probabilities of the sidechains to the scaffold. The possible sets of positions on the scaffold were defined as configuration of scaffolds, and were denoted by *N*_*r*_ in this study. G.A.M.E. uses the configurations of the selected scaffold by linking possible side chains to elucidate suitable chemical structures corresponding to the input molecular weights of a mixture. All datasets are available on GitHub at https://doi.org/10.5281/zenodo.237579. After dataset curation, only 26,641 scaffold files are suitable for the following test with *w*_*max*_ = 500, with total configuration number of 51,562.

In addition, for comparisons with the CPU DP algorithm in real study cases, verified constitutions from four natural products (*Cuscuta chinensis*, *Ophiopogon japonicus*, *Polygonum multiflorum* and *Angelica* sp.) obtained from the Natural Product Laboratory of Taiwan Medical and the Pharmaceutical Industry Technology and Development Center were used as testing datasets. The numbers of verified constitutions in the *Cuscuta chinensis*, *Ophiopogon japonicus*, *Polygonum multiflorum*, and *Angelica* sp. datasets were 5, 7, 7, and 45, respectively; the corresponding molecular ranges were from 286.24 to 478.41, from 328.32 to 370.36, from 270.24 to 578.53 and from 162.03 to 574.29, respectively.

### GPU configuration

Computations were performed on an ASUS^®^ ESC4000 G2 Server with two Intel^®^ Xeon E5-2630 v2 processors (3.10 GHz) running under CentOS 6.7. We used Python 2.7 and CUDA 7.5 in combination with PyCuda 2017.1. Our graphic card was an NVIDIA^®^ Tesla K40c with a compute capability of 3.5, equipped with 15 streaming multiprocessors of 192 cores each, 11,439 MB of global memory and 49 kB of shared memory. The computational resources were provided by Computer and Networking Center, National Taiwan University.

### Problem definitions

Before introducing the GPU algorithm in later sections, we first defined notations used in the algorithms, and illustrated the problem of structural elucidation in this section.

#### Input data notations (n, w_min_, w_max_, D, R, K, h, W, P)

The algorithm takes as input nine parameters: six scalars and three matrices. They are defined as follows: *n* ∈ ℕ, *w*_*min*_ ∈ ℝ^+^, *w*_*max*_ ∈ ℝ^+^ (*w*_*max*_ ≥ *w*_*min*_), *D* ∈ ℕ, *R* ∈ ℕ, *K* = {*k*_*0*_, *…*, *k*_*n*−1_} ∈ ℕ^*n*^, *h* = *max*(*K*), *W* = {*w*_*s,j*_}_(*s*,*j*)∈[|*0,n*|]×[|*0,h*−1|]_ ⊂ (ℝ^+^)^*h*×*n*^ and *P* = {*p*_*s,j*_}_(*s,j*)∈[|0*,n*|]×[|0*,h*−1|]_ ⊂ [0,1]^*h*×*n*^. Moreover, we define ∀*s*∈[|0*,n*|], *j* ≥ *k*_*s*_ ⇒ *p*_*s,j*_ = *w*_*s*,*j*_ = 0. All necessary information in a scaffold used in G.A.M.E. is represented by (*n*, *K*, *W*, *P*). *n* is the number of possible atoms (positions) in the scaffold that can be linked by side chains. *K* = {*k*_*0*_, *…*, *k*_*n*−1_} contains the sidechain counts, defined such that *k*_*s*_ is the number of possible sidechains that can be extended at a position *s* in the scaffold. *W* = {*w*_*s,j*_}_(*s*,*j*)_ represents molecular weights of the possible extended sidechains, defined such that *w*_*s,j*_ is the weight of the *j*th sidechain at position *s*. *P* = {*p*_*s,j*_}_(*s,j*)_ denotes probabilities of the extended sidechains to the scaffold, defined such that *p*_*s,j*_ is the probability of the *j*th sidechain linked at position *s* of the scaffold. Given a scaffold, *S* = (*n*, *K*, *W*, *P*), Ψ(*S*) = [|0, *k*_*0*_ − 1|] × … × [|0, *k*_*n*−1_ − 1|] is defined as a set of all structures that can be generated by extending the possible sidechains on this scaffold. Furthermore, Ψ_s_(*S*) = [|0, *k*_*0*_ − 1|] × … × [|0, *k*_*s*_ − 1|] denotes a set of all structures that can be generated on the sub-scaffold *S*_*s*_, containing only the first *s* substituted positions of the scaffold *S* (from position *0* to *s*). Given a compound *x* ∈ Ψ(*S*), the *mass* of *x* is designated as *g*(*x*) = Σ *w*_*s,j*_, and the probability of *x* that was generated by extending sidechains to the scaffold *S* is defined by *f*(*x*) = Π *p*_*s,j*_. (*w*_*min*_, *w*_*max*_, *D*) represents a set of *mass* information. [*w*_*min*_, *w*_*max*_] is the interval of masses corresponding to the *mass* peak from the MS experiment in a mixture. This interval depends on the resolution of the mass spectrometer. *D* is the number of *mass* decimal digits available for *w*_*min*_ and *w*_*max*_. In the DP algorithm [20] for structure elucidation, the molecular weights (*mass*) has to be converted to a value of integers format multiplied by 10^*D*^. The converted value of mass will lead to no loss of significant digits in the DP algorithm.

#### Optimization problem

The processes of structure elucidation in a mixture can be represented by an optimization problem. In G.A.M.E., the definition of the optimization problem is same with the CSCCP [20]. Given a scaffold *S* = (*n*, *K*, *W*, *P*) and a mass peak M = (*w*_*min*_, *w*_*max*_, *D*), the CSCCP is to find the top *R* most probable compounds in Ψ(*S*) having weights inside the interval [*w*_*min*_, *w*_*max*_]. We have noted that a scaffold might contain different configurations in “Datasets” section. In one CSCCP, we only manipulate one configuration of the scaffold. The CSCCP is to search optimal chemical structures, *x* ∈ Ψ(*S*), that extend the possible substituents to a configuration of the given scaffold while maximizing the objective function, *f*(*x*), such that two constraints, *x* ∈ Ψ(*S*) and *g*(*x*) ∈ [*w*_*min*_, *w*_*max*_], are fulfilled. The R compounds, *x* ∈ Ψ(*S*) having highest values of *f*(*x*), and containing total weights between *w*_*min*_ and *w*_*max*_ are regarded as the best predicted structures in a CSCCP. The previous studies [20] used the DP algorithm to solve the optimization problem, and we modified the DP algorithm based on the GPU parallelism.

#### Transformed input ($w_{min}^{\prime}$, $w_{max}^{\prime}, W^{\prime}$)

Because the generated structures must be indexed by its weights in the design of DP algorithm, all *mass* values must be converted to integers by multiplying 10^*D*^ in the program. We therefore define $w_{min}^{\prime}$ = *w*_*min*_ × 10^*D*^ ∈ ℕ, $w_{max}^{\prime}$ = *w*_*max*_ × 10^*D*^ ∈ℕ, and *W*′ = {$w_{s,j}^{\prime}$}_*s,j*_, such that $w_{s,j}^{\prime}$ = *w*_*s,j*_ × 10^*D*^. ($w_{min}^{\prime}$, $w_{max}^{\prime}$, *W*′) is the integer format of a mass peak.

#### Intermediated variables (C, L)

The DP algorithm used two important matrices to traverse a list of selected sidechains, and extend the sidechains to the given scaffold during the process of generating possible structures in a mixture: the cost matrix *C* = {*c*_*s,w,r*_}_*s,w,r*_ ∈ $\left[ {0,1} \right]^{{\left( {n \times w_{max}^{\prime} \times R} \right)}}$ and the sidechain information matrix *L* = {*l*_*s,w,r*_}_*s,w,r*_ ∈ $\left[ {\left| {0,h} \right|} \right]\left[ {\left| {0,R} \right|} \right]^{{\left( {n \times w_{max}^{\prime} \times R} \right)}}$, with (*s,w,r*) ∈ [|0,*n*|] × [|0, $w_{max}^{\prime}$ |] × [|0,*R*|]. The value of *c*_*s,w,r*_ represents the probability of the *r*th highest value of *f*(*x*) when only *s* out of *n* substituted positions on a given scaffold *S* has been traversed by the DP program, and the *mass* of compounds in Ψ_s_(*S*) generated by the selected sidechains linking on the *S* is equal to *w*÷10^*D*^ as well. *w* denotes the molecular weight ranging from 0 to $w_{max}^{\prime}$ in integer format. The DP algorithm iteratively evaluates the possible extended sidechains on scaffold *S* from position 0 to *s*, and calculates the values in *C* for all the molecular weight from 0 to $w_{max}^{\prime}$ and *R* values from 0 to *R* for each *s* substituted positions. When all the values in *C* have been calculated by the DP algorithm, the generated compounds with the values of *c*_*n,w,r*_, where *w* ∈ [$w_{min}^{\prime}$, $w_{max}^{\prime}$], and *r* ∈ [0,*R*], are the optimal predicted results. The *l*_*s,w,r*_ is the tuple $\left( {k_{s,w,r} ,r_{s,w,r}^{prev} } \right)$, where *k*_*s,w,r*_ is a sidechain index at position *s* of the compound in Ψ_s_(*S*) with *r*th highest probability and total weight *w*, and $r_{s,w,r}^{prev}$ is the rank of the compound in Ψ_s-1_(*S*) with the total weight $w - w_{{s,k_{s,w,r} }}^{\prime}$ that was used to construct the *r*th compound in Ψ_s_(*S*). *l*_*s,w,r*_ were used to traverse a list of selected sidechains generating the *c*_*s,w,r*_ in the DP algorithm. The detailed DP algorithm is introduced in the next section.

### GPU algorithm implementation

To solve the CSCCP, the DP algorithm that was applied in our GPU system can be implemented in the following two phases.

*First phase*: the first phase comprises four imbricated loops indexed by (*s*,*w*,*r*,*k*). For a loop state, (*s,w,r*) ∈ [|0,*n*|] × [|0, $w_{max}^{\prime}$|] × [|0,*R*|], we have *k* ∈ [|0,*k*_s_-1|]. In the first loop, the algorithm evaluates the values of *C* from position 0 to *s* on the given scaffold. At each iteration on *s*, the algorithm computes all the values *c*_*s*,:,:_ = {*c*_*s,w,r*_|(*w,r*) ∈ [|0, $w_{max}^{\prime}$|] × [|0,*R*|]}. The second loop is the traverse of all possible weight *w* from 0 to $w_{max}^{\prime}$. This aim is achieved as follows. For each possible weight *w*, the next (*k*,*r*) loops compute the set $Tmp = \left\{ {\left( {k,r,p_{s,k} \times c_{{s - 1,w - w^{\prime}_{{s,k_{s,w,r} }} }} } \right)} \right\}_{k.r}$. The set is then sorted according to the updated probability in the 3rd component of *Tmp*. Let (*T*_*0*_,*T*_1_,…,*T*_*R*-1_) be the highest *R* ranked elements for the *Tmp* calculation with the following notation: ∀*r* ∈ [|0,*R*|], *T*_*r*_ = (*t*_*0*_^(r)^, *t*_*1*_^(r)^, *t*_*2*_^(r)^), *c*_*s,w,r*_ = *t*_*2*_^(r)^ and *l*_*s,w,r*_ = (*t*_*0*_^(r)^,*t*_*1*_^(r)^). Then, *T*_*r*_ is used to update *C* and *L* in each iteration.

This phase is the main computational part of the DP algorithm. Because the loop on *w* goes from *0* to $w_{max}^{\prime}$ = *w*_*max*_ × 10^*D*^, the complexity of the algorithm is proportional to 10^*D*^; for each additional decimal digit required, the running time extends ten times longer.

*Second phase*: the second phase outputs the final ranking of the generated structures in the first phase corresponding to the mass peak. The top *R* probabilities are simply the top *R* elements in the set {*c*_*n,w,r*_|(*w*,*r*) ∈ [|$w_{min}^{\prime}$, $w_{max}^{\prime}$|] × [|0,*R*|]}. Because of a simple recursive backtracking algorithm, the matrix *L* can be used to compute the compound corresponding to each probability of the above top *R* probabilities. This phase is not computationally demanding and does not require acceleration.

In the next sections, global memory and shared memory requirements for the DP algorithm [20] are analyzed. The memory usage is reduced by discarding unnecessary data after each iteration and applying a compression technique. Different parallelization schemes were then designed, and the best scheme was applied according to parameter constraint assessment (see Additional file 1). We evaluated those schemes on the basis of global memory requirements, shared memory requirements, memory access patterns and the amount of parallelism. For the experimental data analysis section, we compared the difference between the performance of our GPU algorithm and that of the previous CPU version.

#### Memory usage reduction

Given that the sizes of *C* and *L* matrix are proportional to 10^*D*^ (as well as *n* and *r*), they become too large to fit in GPU memory for larger values of *D*. In fact, only certain values of *C* are required to be accessible at any time during the execution of the algorithm. At a loop state *s*, only the sub matrices c_*s*-1,:,:_ and *c*_*s*,:,:_ are used to update the matrix *C*; we denote these two matrices as $C^{ - } = \left\{ {C_{w,r}^{ - } } \right\}_{w,r}$ and $C^{ + } = \left\{ {C_{w,r}^{ + } } \right\}_{w,r}$ respectively. At the iteration on position *s*, the size of *C* ^−^ and *C* ^+^ are each $w_{max}^{\prime}$ × *R*. Since we used the *C* ^−^ and *C* ^+^ to replace *C*, we only required 2 × $w_{max}^{\prime}$ × *R* values instead of *n* × $w_{max}^{\prime}$ × *R*, thus representing 100(1 − 2/*n*)% of saved memory. Because the number of bytes per floating type value is 4, 8 × $w_{max}^{\prime}$ × *R* bytes of global memory are used to store *C*. For a scaffold with 10 substituted positions, 80% of the memory can be reduced. On the other hand, all values of *L* from *s* = *0* to *n* must be kept in the device memory until the end of the execution because *L* must be traversed to retrieve the lists of the selected sidechains for generation of compounds having the highest probability values in *C*. To reduce memory usage, we designed a compression technique. We created a compressed matrix *L* ^*c*^, denoted $L^{c} = \left\{ {l_{w,r}^{c} } \right\}_{w,r}$, defined by $\forall \left( {w,r} \right),l_{w,r}^{c} = \overline{{l_{1,w,r} }} \cdot \overline{{l_{2,w,r} }} \cdots \overline{{l_{n - 1,w,r} }} \cdot \overline{{l_{n,w,r} }}$, where $\overline{{l_{s,w,r} }}$ refers to the binary code of *l*_*s,w,r*_, and denotes the string concatenation. For a given value (*w,r*), since each of *k*_*s,w,r*_ and $r_{s,w,r}^{prev}$ is declared as 16-bits integer in DP algorithm, we totally require *n* × *32*-bits integer to store *n* values of *k*_*s,w,r*_ and $r_{s,w,r}^{prev}$ without compression. In G.A.M.E., we merged totally *n* values of $\left( {k_{s,w,r} ,r_{s,w,r}^{prev} } \right)$ (*s* = 0 to *n* for a given (*w,r*)) into two 32-bits integers. The number of bits required to store *k*_*s,w,r*_ and $r_{s,w,r}^{prev}$ are ⌊*log*_2_(*k*_*s*_) + 1⌋ and ⌊*log*_2_(*R*) + 1⌋, respectively. By summation of the total *n* values of bits for $\left( {k_{s,w,r} ,r_{s,w,r}^{prev} } \right)$, this compression technique were then be limited to the constraints described in Eqs. 1 and 2. After using the *L* ^*c*^ compression technique, only two 32-bit integers are used for each $l^{{_{w,r}^{c} }}$. Therefore, only 8 × $w_{max}^{\prime}$ × *R* bytes of global memory are needed to store *L* instead of 8 × *n* × $w_{max}^{\prime}$ × *R* bytes. In this case, 100 (1 − 1/*n*)% memory is saved.

1 $$\mathop \sum \limits_{{{\text{s}} = 0}}^{{{\text{n}} - 1}} { \lfloor }log_{2} \left( {{\text{k}}_{\text{s}} } \right) + 1{ \rfloor } \le 32$$

2 $$n{ \lfloor }log_{2} \left( R \right) + 1{ \rfloor } \le 32$$

#### Parallelization on the GPU

**Fig. 1:**
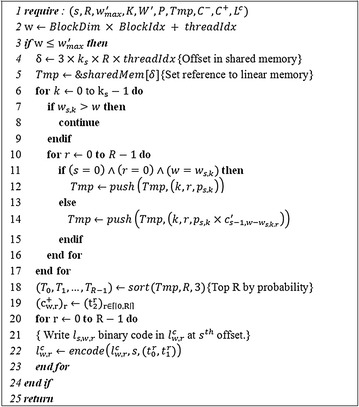
The *Algorithm 1 Kernel*. The *sharedMem* variable is a pointer to the dynamically allocated shared memory. The *push*(*X,y*) function appends element *y* to array *X* and returns *X*. *sort*(*X,R,i*) returns the top *R* elements when the elements of *X* are sorted by their *i*th component. *encode*(*X,s,y*) writes the binary code of *y* at the *s*th offset of $X\left( {slog_{2} \left( R \right)} + \sum\nolimits_{i = 0}^{n} {{ \lfloor }log_{2} \left( {k_{i} } \right) + 1{ \rfloor }} \right)$ and returns *X*

According to the iterative nature of the dynamic programming, it is not possible to parallelize over *n* in the first loop of the DP algorithm. Nevertheless, it would be possible to parallelize the DP program in the following designs: (*a*) have each block handle a range of *w* values, and have each thread in this block process a *w* value, (*b*) have each block handle a *w* value, and have each thread in this block process a *k* value, or (*c*) have each block handle a *w* value, and have each thread in this block process a *r* value. In this study, we implemented the GPU parallelization based on the design (*a*) because it enabled the highest degree of parallelism. According to design (*a*), the pseudocode of our DP CUDA kernel is provided in *Algorithm 1* (Fig. 1). Each thread with thread index (*threadIdx*) processes *Algorithm 1*, and the loops for (*k,r*) ∈ [|0,*h*|] × [|0,*R*|] are implemented sequentially from lines 6 to 17. *BlockDim* is the number of threads per block, and *BlockIdx* represents the Block index of the processing thread on *threadIdx*. Thus, the code in line 2 evaluates the required *mass* (molecular weight) that is processed by the thread on *threadIdx*. *C* ^−^, *C* ^+^ and *L* ^*c*^ are allocated in the GPU global memory, whereas *K*, *W*′, *P* are stored in GPU Constant memory (i.e., the constant memory provides a cached access to constants). The pseudocode from lines 18 to 22 is similar to Su’s DP [20], which uses a sort algorithm to retrieve the top R values of *T*_*r*_ and updates the *C* and *L* matrices. The parameter limitations in the G.A.M.E. were described in the Additional file 1.

## Results and discussion

### Parameter tuning of GPU computing

Before exploiting the advantages of GPU computing, several parameters must be determined to ensure the optimal performance of G.A.M.E. We preliminarily analyzed our datasets and inferred the optimal values of *R*, *D* and *w*_*max*_, which are all related to the memory usage and time complexity of G.A.M.E. Among them, *R* is the output number of structures, *D* is the number of decimal digits of mass for computing, and *w*_*max*_ is the maximal total molecular weight of extended side chains on a given scaffold. On the basis of the 83,242 scaffold files, the average number of summation of the molecular weights from all possible substituents in each compound was 89.42, and the average number of maximum possible sidechains of scaffolds, *h*, was 4. Additionally, the average number of required ranks, *R*, that matched the verified searched ground truth data among the four testing datasets was 8. To calculate the top R solutions of a given scaffold, the CSCCP program outputs R × *N*_*r*_ solutions, where *N*_*r*_ is the number of configurations in one given scaffold; on average, *N*_*r*_ is 5. According to the previous studies [20], the value of *R* in 3 is sufficient to identify the correct structures in four testing datasets. The number of decimal digits, *D*, with a value of 5 is also sufficient to produce the real optimal solutions among the four testing cases. Therefore, the values 3, 5, and 500 for *R*, *D*, and *w*_*max*_, respectively, were adequate to solve the CSCCP using our algorithm.

### Performance of GPU-based G.A.M.E. compared with that of CPU DP

**Fig. 2:**
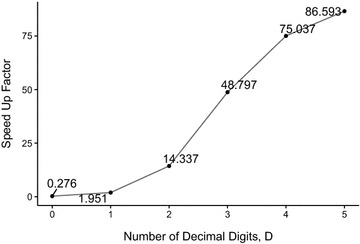
Acceleration of G.A.M.E. relative to Su’s CPU DP as a function of the number of decimal digits, *D*. Each data point is obtained by averaging the running time of 1713 suitable scaffolds from the NPDBs. Fixed parameters: *ThreadsPerBlock* = 64

**Fig. 3:**
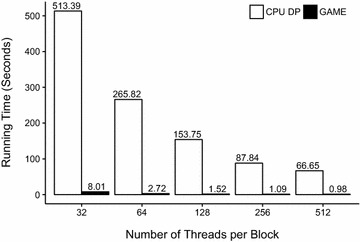
Running time of G.A.M.E. compared with Su’s CPU DP. Each bar was obtained by averaging the running time of the 500 different randomly selected scaffold configurations from the NPDBs, sharing the same number of threads per block. Fixed parameters: *D* = 5, *w*_*max*_ = 500

To compare the performance of our G.A.M.E. algorithm with Su’s original CPU DP algorithm, analyses were performed on NPDB, with different *D* and number of threads per block in GPU kernel, as shown in Figs. 2 and 3. Figure 2 illustrates that the acceleration of G.A.M.E. with respect to CPU DP becomes increasingly significant as *D* increases and is the most significant at *D* = 5. The execution time was calculated from averaging the running time of 1713 suitable scaffolds from the NPDB. The parameters of *ThreadsPerBlock*, *w*_*max*_, and *R* are set to 64, 500 and 3, respectively. As expected, the performance of G.A.M.E. grew with the number of decimal digits, *D*, because the amount of computation is proportional to 10^*D*^. By increasing the value of *D*, the amount of parallelism was maximized. When *D* = 0, the GPU was under-utilized, thus leading G.A.M.E. to be slower than the CPU DP. The average running time of G.A.M.E. was 0.0108 s when *D* = 0, whereas the CPU DP average running time was 0.0029 s. When D = 5, the average running time of G.A.M.E. was 1.54 s. By comparison, CUP DP had an average running time of 146.77 s. In fact, the running time of CPU DP dramatically increased, whereas G.A.M.E. remained within an appropriate time when *D* increased. Overall, G.A.M.E. showed a successful hardware-accelerated performance increase relative to the performance of Su’s original DP algorithm. Furthermore, since the accuracy of the DP algorithm has been demonstrated by Su [20], and we did not change the core of the original DP algorithm [20] in the G.A.M.E. algorithm, our system must maintain the same accuracy.

Whereas Fig. 2 focused on the effect of the number of decimal digits, *D*, on performance, Fig. 3 shows that G.A.M.E. is stable in every case and provides over 64-fold acceleration when the number of threads per block in GPU kernel is 32. In the GPU programming environment, a GPU kernel launches a grid of thread blocks to attain parallel computing. The number of threads in each thread block was denoted by *ThreadsPerBlock* in this study. *ThreadsPerBlock* will dominate both the time performance and memory limitation of the computation. (Detailed definition and relation of blocks, threads, and memory in GPU kernel was described in Additional file 1). For evaluation of time performance with different *ThreadsPerBlock in G.A.M.E.,* the average running time in seconds was calculated by averaging the running time with samplings of 500 randomly chosen scaffold configurations among the NPDB for cases with 32, 64, 128, 256 and 512 threads per block. In all cases with more than 32 threads per block, the running time was below 3 s, whereas Su’s CPU DP ran in 144 s on average. Among these cases, we obtained an average acceleration factor of 82.38. Therefore, we can expect that for compounds with higher *h* and a larger *w*_*max*_, the DP algorithm required longer execution time to solve CSCCP, whereas G.A.M.E. continued to execute in relatively lower running time. Given the different *ThreadsPerBlock*, the average execution time of G.A.M.E. were all similar, stable, and sufficiently short to solve CSCCP in practical cases because of the successful parallel computations.

### Optimal number of mass decimal digits for G.A.M.E.

To determine the applicability of our G.A.M.E. implementation, we first performed a series of tests to estimate what proportion of the NPDB scaffolds could be used as an input for G.A.M.E. We listed the different reasons causing the CSCCP to be non-feasible and evaluated the proportions of unique non-feasible cases. Table 1 illustrates the percentage of feasible cases within all configurations of scaffolds of the NPDB for a target weight $w_{max}^{\prime}$ = 500 × 10^*D*^ and with the value of *D* increasing from 0 to 7. The feasible cases indicate the configurations of given scaffolds that could be performed well in G.A.M.E., adhering to all hardware-imposed constraints. The proportions were obtained by analyzing 51,562 suitable configurations of all 83,242 scaffolds of the NPDB when *w*_*max*_ = 500. We found that 99.28% of the cases were feasible for *D* ≤ 5. For *D* = 6, only 32.94% of the cases were feasible, and for *D* = 7, the proportion of feasible cases was 1.06%. In other words, cases with *D* values larger than 6 are beyond the limit of G.A.M.E. Nevertheless, the previous studies [20] showed that a value of *D* = 5 is sufficient to identify the correct structures in four testing datasets. It is unnecessary to waste the memory space and delay the execution time by using too many decimal digits, because statistically real solutions can still be output when *D* is less than 6.

**Table 1:** The percentage of feasible cases within all configurations of scaffolds of the NPDBs

Number of decimal digits, *D*	≤5	6	7
Proportion of feasible cases (%)	99.28	32.94	1.06

The statistics were obtained from all scaffolds of the NPDBs. Fixed parameters: *w*_*max*_ = 500

We then analyzed the reason why some cases are not feasible, using the case in which *w*_*max*_ = 500. Table 2 illustrates the different types of problems distributed among non-feasible cases. Most of the cases were not feasible when *D* ≥ 6 because the usage of total global memory exceeded maximal resource of the system. Furthermore, since the design of compression technique (described in “Memory usage reduction” section) for memory reduction in G.A.M.E. was limited in Eq. 1 and 2, the compression restraints made 369 configurations were not feasible in all cases of *D*.

**Table 2:** Statistics on non-feasible cases

Configurations with specific problem types	D ≤ 5	D = 6	D = 7
Insufficient global memory (Supp. Eq. 1)	0	34,209	50,646
Compression impossible (Eqs. 1 and 2)	369	0	0
Both problems	0	369	369
Total	369	34,578	51,015

The statistics were obtained on the non-feasible configurations of Table 1. Fixed parameters: *w*_*max*_ = 500

### Hardware-imposed limitations

By means of hardware acceleration to speed up the CPU DP algorithm for CSCCP, certain parameters of the developed G.A.M.E. suffered from hardware-imposed constraints. Table 3 lists three types of constraints of the observed resources for different parameters on the NVIDIA^®^ Tesla K40c. The first row in Table 3 shows the evaluated maximum molecular weight (*w*_*max*_) for different values in *R* and *D*, according to an assessment of the parameter constraints (see Additional file 1: Eq. 3). The *w*_*max*_ must be less than or equal to 2499 (Da) for *R* = 3, compared with 749 (Da) for *R* = 10. The total required global memory based on different *R* values when *D* = 5 and *w*_*max*_ = 500 is listed in the second row of Table 3. Whenever the value of *R* is 3 or 10, the required memories (2.24 GB or 7.45 GB) are still under the capacity of the 11,440 MB global memory of the NVIDIA^®^ Tesla K40c. From the parameter constraint assessment (See Additional file 1: Eq. 4), the possible maximum number of threads per block for parallel computation given by different *R* and *h* are shown in the third row of Table 3. Lower values of *R* and *h* correspond to a higher number of threads per block that the algorithm can execute for parallelization. To ensure stable computing, our algorithm automatically calculates the possible maximum number of threads per block according to the parameters *R* and *h*. If there are cases that exceed the constraint of the maximum molecular weight (*w*_*max*_ = 500) and the compression technique of Eqs. 1 and 2, the system ignores those non-feasible calculations.

**Table 3:** Parameter constraints on the NVIDIA^®^ Tesla K40c

Resources	Constraints	Parameters
Target weight (*w*_*max*_)	≤2499 Da	*R* = 3, *D* = 5
	≤749 Da	*R* = 10, *D* = 5
Memory usage	2.24 GB	*R* = 3, *D* = 5, *w*_*max*_ = 500
	7.45 GB	*R* = 10, *D* = 5, *w*_*max*_ = 500
Number of threads per block (*ThreadsPerBlock)*	≤512	*R* = 2, *h* = 4
	≤256	*R* = 3, *h* = 5
	≤128	*R* = 3, *h* = 10
	≤64	*R* = 6, *h* = 10
	≤32	*R* = 10, *h* = 10

### Acceleration validation in real cases

**Fig. 4:**
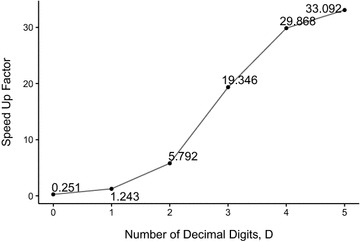
Acceleration of G.A.M.E. relative to Su’s CPU DP as a function of the number of decimal digits, *D*. Each data point is obtained by averaging the running time of 200 randomly sampled scaffolds and molecular mass combinations from the four natural products

In order to validate the accelerated G.A.M.E. system, we used four testing sets from real analysis data of four natural products: *Cuscuta chinensis*, *Ophiopogon aponicas*, *Polygonum multiflorum*, and *Angelica sp.* The execution time was calculated from averaging the running time of 10 random samplings of 20 cases in each sampling within these four testing datasets. The *R* parameter was set to 3; the *w*_*max*_ parameter was from molecular weights detected from real MS experiments; and the *ThreadsPerBlock* parameter was automatically calculated with the most blocks feasible calculated by the parameter assessment equation (see Additional file 1: Eq. 4). The speed up factors, the CPU DP average running time divided by GPU running time, was illustrated in Fig. 4. In this testing analysis, the GPU was under-utilized when D = 0. The average running time of G.A.M.E. was 0.0207 s when *D* = 0, whereas the CPU DP average running time was 0.0047 s. Although G.A.M.E. cannot show the improved performance when *D* = 0, the GPU programming showed the significant accelerations when *D* is greater than 0. When D = 5, the average running time of G.A.M.E. was 20.72 s. By comparison, CUP DP had an average running time of 324.16 s. As a result, in real cases that *w*_*max*_ values were set from real experiments, the G.A.M.E. also dramatically accelerated the performance compared with Su’s original DP algorithm. In this section, we have sufficiently demonstrated that G.A.M.E. outperformed the process of the CSCCP problem in real cases, compared with the original DP algorithm.

## Conclusions

Our G.A.M.E. method is executed in silico to predict the constituents of a mixture on the basis of its mass spectral data by exploiting a scaffold database with sidechain probability information, such as Harn’s Natural Product Scaffold database. By harnessing the power of modern GPU graphic cards, G.A.M.E. outperforms Su’s DP algorithm with an average acceleration factor of 33 in 99% of the cases. It can be applied to situations in which up to 5 decimal digits are required. Our studies demonstrate three advantages in using a GPU-accelerated algorithm to solve CSCCP: (a) the algorithm can be implemented easily, (b) CSCCP can be appropriately divided into many small tasks to perform parallel computations, and (c) a stable running time is achieved. We conclude that our hardware-accelerated algorithm is an excellent method to solve CSCCP. As expected, with the rapid development of PC hardware, the use of GPU acceleration or even the application of distributed systems will be a practical way to directly and efficiently identify the components of an unknown mixture.

## Additional file

**Additional file 1.** Contains the supplementary information of the manuscript. This includes (1) overview of GPU programming framework with CUDA; (2) parameter limitations assessment imposed by hardware; (3) an introduction for the prediction procedure in NP-StructurePredictor; (4) an introduction of dynamic algorithm for structure elucidation.

## Abbreviations

G.A.M.E.: GPU-accelerated mixture elucidator
DP: dynamic programming
CUDA: compute unified device architecture
MS: mass spectrometry
NMR: nuclear magnetic resonance
CASE: computer-aided structure elucidation
NPDB: natural products database
CSCCP: chemical substituent core combinatorial problem
VGA: video graphics array

## References

[1] Harvey AL, Edrada-Ebel R, Quinn RJ (2015). The re-emergence of natural products for drug discovery in the genomics era. Nat Rev Drug Discov.

[2] Kim HK, Choi YH, Verpoorte R (2010). NMR-based metabolomic analysis of plants. Nat Protoc.

[3] Corcoran O, Mortensen RW, Hansen SH, Troke J, Nicholson JK (2001). HPLC/1H NMR spectroscopic studies of the reactive alpha-1-O-acyl isomer formed during acyl migration of S-naproxen beta-1-O-acyl glucuronide. Chem Res Toxicol.

[4] Corcoran O, Spraul M (2003). LC-NMR-MS in drug discovery. Drug Discov Today.

[5] van der Hooft JJJ, Mihaleva V, de Vos RCH, Bino RJ, Vervoort J (2011). A strategy for fast structural elucidation of metabolites in small volume plant extracts using automated MS-guided LC-MS-SPE-NMR. Magn Reson Chem.

[6] Kind T, Fiehn O (2010). Advances in structure elucidation of small molecules using mass spectrometry. Bioanal Rev.

[7] Elyashberg ME, Gribov LA (1968). Formal-logical method for interpreting infrared spectra from characteristic frequencies. J Appl Spectrosc.

[8] Lederberg J, Sutherland GL, Buchanan BG, Feigenbaum EA, Robertson AV, Duffield AM (1969). Applications of artificial intelligence for chemical inference. I. The number of possible organic compounds. Acyclic structures containing C, H, O, and N. J Am Chem Soc.

[9] Nelson DB, Munk ME, Gash KB, Herald DL (1969). Alanylactinobicyclone. Application of computer techniques to structure elucidation. J Org Chem.

[10] Sasaki S, Abe H, Ouki T, Sakamoto M, Ochiai S (1968). Automated structure elucidation of several kinds of aliphatic and alicyclic compounds. Anal Chem.

[11] Christie BD, Munk ME (1991). The role of 2-dimensional nuclear-magnetic-resonance spectroscopy in computer-enhanced structure elucidation. J Am Chem Soc.

[12] Peng C, Yuan SG, Zheng CZ, Hui YZ (1994). Efficient application of 2D NMR correlation information in computer-assisted structure elucidation of complex natural-products. J Chem Inf Comput Sci.

[13] Lindel T, Junker J, Kock M (1999). 2D-NMR-guided constitutional analysis of organic compounds employing the computer program COCON. Eur J Org Chem.

[14] Blinov KA, Carlson D, Elyashberg ME, Martin GE, Martirosian ER, Molodtsov S (2003). Computer-assisted structure elucidation of natural products with limited 2D NMR data: application of the StrucEluc system. Magn Reson Chem.

[15] Elyashberg ME, Blinov KA, Williams AJ, Molodtsov SG, Martin GE, Martirosian ER (2004). Structure elucidator: a versatile expert system for molecular structure elucidation from 1D and 2D NMR data and molecular fragments. J Chem Inf Comput Sci.

[16] Elyashberg ME, Williams A, Martin GE (2008). Computer-assisted structure verification and elucidation tools in NMR-based structure elucidation. Prog Nucl Magn Reson Spectrosc.

[17] Butler MS (2004). The role of natural product chemistry in drug discovery. J Nat Prod.

[18] Elyashberg M, Blinov K, Molodtsov S, Williams A (2012). Elucidating ‘undecipherable’ chemical structures using computer-assisted structure elucidation approaches. Magn Reson Chem.

[19] Harn Y-C (2011) Structure hunter: prediction of novel chemical structures in a mixture [Master Dissertation]. Taipei, Taiwan: National Taiwan University

[20] Su B-H (2012) A chemical substituents-core combinatorial optimization and hERG toxicity prediction in drug design [Ph.D. Dissertation]. Taipei, Taiwan: National Taiwan University

[21] Owens JD, Houston M, Luebke D, Green S, Stone JE, Phillips JC (2008). GPU computing. Proc IEEE.

[22] Asanovic K, Bodik R, Demmel J, Keaveny T, Keutzer K, Kubiatowicz J (2009). A view of the parallel computing landscape. Commun ACM.

[23] Owens JD, Luebke D, Govindaraju N, Harris M, Kruger J, Lefohn AE (2007). A survey of general-purpose computation on graphics hardware. Comput Graph Forum.

[24] Miao Y, Sinko W, Pierce L, Bucher D, Walker RC, McCammon JA (2014). Improved reweighting of accelerated molecular dynamics simulations for free energy calculation. J Chem Theory Comput.

[25] Clark AJ, Tiwary P, Borrelli K, Feng S, Miller EB, Abel R (2016). Prediction of protein-ligand binding poses via a combination of induced fit docking and metadynamics simulations. J Chem Theory Comput.

[26] Gowthaman R, Lyskov S, Karanicolas J (2015). DARC, 2.0: improved docking and virtual screening at protein interaction sites. PLoS ONE.

[27] Galindo-Murillo R, Roe DR, Cheatham TE (2014). On the absence of intrahelical DNA dynamics on the μs to ms timescale. Nat Commun.

[28] Chen W, Zhu Y, Cui F, Liu L, Sun Z, Chen J (2016). GPU-accelerated molecular dynamics simulation to study liquid crystal phase transition using coarse-grained gay-berne anisotropic potential. PLoS ONE.

[29] Johnson DK, Karanicolas J (2016). Ultra-high-throughput structure-based virtual screening for small-molecule inhibitors of protein–protein interactions. J Chem Inf Model.

[30] Korpar M, Šošić M, Blažeka D, Šikić M (2015). SW# db: GPU-accelerated exact sequence similarity database search. PLoS ONE.

[31] Tyzack JD, Mussa HY, Williamson MJ, Kirchmair J, Glen RC (2014). Cytochrome P450 site of metabolism prediction from 2D topological fingerprints using GPU accelerated probabilistic classifiers. J Cheminform.

[32] (2010) The Dictionary of Natural Products database is available from Chapman & Hall/CRC. http://dnp.chemnetbase.com/ [updated 2017; cited 2017]

[33] Irwin JJ, Shoichet BK (2004). ZINC—a free database of commercially available compounds for virtual screening. J Chem Inf Model.

[34] Chen CY-C (2011). TCM Database@Taiwan: the world’s largest traditional Chinese medicine database for drug screening in silico. PLOS One.

