# Supplementary material for: G.A.M.E.: GPU-accelerated mixture elucidator
Source: J Cheminform. 2017 Sep 15;9:50. doi: 10.1186/s13321-017-0238-7 (PMC5602814; doi:10.1186/s13321-017-0238-7)
Supplement: Supplementary file 1 — Additional file 1. Contains the supplementary information of the manuscript. This includes (1) overview of GPU programming framework with CUDA; (2) parameter limitations assessment imposed by hardware; (3) an introduction for the prediction procedure in NP-StructurePredictor; (4) an introduction of dynamic algorithm for structure elucidation. [file 13321_2017_238_MOESM1_ESM.docx]

**Supplementary Information**

**Overview of GPU programming framework with CUDA:** In GPU accelerated architecture, parallelized data processes are mapped to independent threads. In 3D graphics, pixels and vertices can be mapped to parallel threads on GPU to speed up the rendering. Similarly, many non-graphic related procedures can be also accelerated by data-parallel processing. In 2006, NVIDIA introduced CUDA, a general purpose parallel computing platform providing a simple programming model that enables the programmer to harness the power of the parallel compute engine of NVIDIA Graphic cards to solve general computational problems (1).

When programming with CUDA, a host program, executed by the CPU, will launch the execution of the device code (called kernel in CUDA). This so called kernel will be executed by the GPU. Before calling a kernel, the programmer will use dedicated functions to allocate memory on the device and then copy data from the host memory to the device memory. The memory can be copied from the CPU's DRAM to the GPU's *global memory*, the *constant memory* (cached) or the *texture memory* (optimized for texture fetching).

A kernel launches a *grid of thread blocks*. Threads in a block can be synchronized while threads from different blocks may not communicate. The programming model gives no control over the block scheduling to the programmer: blocks will be distributed statically between streaming multiprocessors by the hardware. A block requiring too much shared memory will simply not be scheduled to run. Data can be shared accessed concurrently by threads inside the same block, using the *shared memory*. Blocks and grids can be 1, 2 or 3 dimensional depending on the application type and blocks will be identified by a structure called *blockIdx,* and the threads identified by *threadIdx*. Each thread can use those structures to know which block they are located in and what is their index. Therefore different threads can have different behavior depending on their given *blockIdx* or *threadIdx*. Each thread has its own private local memory which uses registers. *Registers* are scarce but provide the lowest latency: accessing data in global memory from thread is orders of magnitudes slower than accessing registers or shared memory.

Threads are gathered in groups of 32 threads called *warps*. A warp will be assigned to a warp scheduler. At a given time, all threads in a warp should execute synchronously the same instruction line. If conditional branching happens, the warp scheduler's instruction dispatch unit will serialize the instructions, thus losing the advantage of parallelism: the phenomenon is called thread divergence. To make most of the GPUs computational power, one needs to maximize parallelism *i.e*. the number of threads, in order to hide *latency*. Standard execution latency (when an instruction depends on the result of the previous instruction) typically takes 22 cycles. By contrast, local memory access takes 400 to 800 cycles. When optimizing a piece of code, one needs to maximize the number of active warps by having a large number of threads, and using few registers.

Finally, memory accesses are operated per warp and are managed in transactions. A single transaction accesses 32, 64 or 128 bytes, aligned to their size. All other accesses are split in multiple instructions. Therefore, we try as much as possible to have successive threads read memory from successive addresses.

**Parameter limitations imposed by hardware**

Due to the scarce nature of memory on GPU chips, our implementation will first verify that enough resources are available. First, according to the derivation of used global memory from compression technique we described, the total global memory should be greater than 16×*w’_max_*×*R* bytes (*GlobalMem*>16×*w’_max_*×*R*). By substituting *w’_max_ = w_max_*×10*^D^* and taking the log of both sides, equation 3 is derived and illustrates the precision *D* constraint. Secondly, since the temporary storage *Tmp* needed to be read and accessed very often with relatively random access patterns, we decided to store it in shared memory, to have a minimum latency. In *Algorithm 1*, the lines 5 to 16 proceed to the storage of *h*×*R* couples of two integer numbers (int32) and one floating number (float32). The total shared memory needed is 3×4×*h*×*R=* 12×*h*×*R* bytes. Thus, given the shared memory per block, the number of threads per block was set to be less or equal to the shared memory per block divided by 12×*h*×*R*. This lead to the constraint described in Equation 4. Because each thread needs 3×*h*×*R* 32 bits values to store *Tmp,* the offset in shared memory accessed by each thread is calculated from line 4 to 5 in *Algorithm 1*. Indeed, with increasing precision parameter *D*, the number of masses *w’_max_* grows orders of magnitudes larger, and with it the number of threads. When launching our kernel, the number of threads per block is set to the maximum possible value for the given CSCCP. To simplify performance analysis, the number of threads was set to the closest power of 2 less or equal to ⌊*sharedMem*÷12×*R*×*max*(*K*)⌋ to (*c.f.* Equation 4). The number of blocks required is then adjusted in order to obtain a total number of threads equal to *w’_max_*. Finally, since we used a row-major representation for the *C*^−^ and *C*^+^ matrices, two consecutive threads access the *C* matrices with a stride exactly equal to *R*. To avoid performance degradation, *R* should be small. In fact, according to the previous DP studies, the compounds can be optimally predicted based on the value of R less than 10.

*D*<*log*_10_(*GlobalMem*/(16×*R*×*w_max_*) (3)

*ThreadsPerBlock* ≤ ⌊*SharedMem*/(*12*×*R*×*max*(*K*))⌋ (4)

## An introduction for the prediction procedure in NP-StructurePredictor

To elucidate unknown chemical structures in mixtures, all structures in *NPDBs* were first broke into one major chemical scaffold and several side chains. The definition of scaffolds is according to Bemis et al (2). The side chains are defined as parts of the structures excluding its scaffold. We only included the side chains that are not hydrogen. The probabilities of occurrence for side chains in each position of scaffold were also calculated. For a scaffold with atom-positions {1, 2, … …, *S*}, the probability of occurrence of side chains *x* at atom-position *y* is defined as following:

| $P\left( x_{y} \right)=\frac{F(x_{y})}{N_{y}}$*,* $\text{for an position }y\in\left\{ 1, 2, \ldots\ldots,S \right\}$ | (1) |
| --- | --- |

Where $F(x_{y})$ is the frequency of side chain *x* occurred at position *y* of the scaffold in *NPDBs*, and $N_{y}$ is the total number of possible side chains occurred at position *y* of the scaffold in *NPDBs*. We also analyzed which possible sets of positions on the scaffold can be extended by the side chains. These possible sets of positions on the scaffold are denoted by *atom-position configurations*. We used the *atom-position configurations* to identify unknown chemical structures by linking appropriate side chains.

NP-StructurePredictor utilizes targeted molecular weights (*targeted MW)* calculated from a list of m/z values in LC-MS experiments and a given scaffold as input information to elucidate the chemical structure of individual components matching the *targeted MW* with that scaffold. To generate structures having *targeted MW*, *W_0_*, NP-StructurePredictor applied a branch and bound algorithm to formulate possible chemical structures by linking all possible side chains on the *targeted scaffolds* to match the *targeted MWs* based on the *atom-position configurations*. A combination of possible side chains on a scaffold is denoted by *C* = (*X_1_, X_2_, …,X_S_*), where *X_n_* is a side chain at atom position *n*, and *S* is the number of atom-positions of a specific scaffold. For the considered scaffold, if we aim to find *R* most possible structures in respect to a *W_0_*, the computational problem to elucidate potential structures can be illustrated as following formulas:

| $\text{Find }\text{R}\text{ combinations }{(C}_{1}, C_{2}, \ldots\ldots, C_{R})$ | (2) |
| --- | --- |
| $\text{such that} P\left( C_{1} \right) is maximum, P\left( C_{1} \right)>P(C_{2})\ldots\ldots>P(C_{R})$ | (3) |
| $\text{and }\text{∀}\text{c}\text{ }\text{∈}{(C}_{1}, C_{2}, \ldots\ldots, C_{R}), \sum_{i=1}^{S} MW\left( X_{i} \right)=w$ | (4) |

where *w* is the molecular weight excluded the MW of the scaffold from *W_0_*, $MW\left( X_{i} \right)$ represents the molecular weight of side chain, $X_{i}$, and the probability of a combination *C* is defined as:

| $P\left( C \right)=\prod_{i=1}^{S} P(X_{i})\text{,} C=(X_{1},X_{2},\ldots\ldots,X_{S})$ | (5) |
| --- | --- |

Probability of a side chain *X_i_* is defined in equation (1). The formula (3) ensures that the combinations of side chains are the best *R* candidates with the highest probability of occurrence in nature, and the formula (4) verifies that the total molecular weight of the selected side chains matches the *w*. The probability of the selected combination of side chains is defined in the equation (5). Because most of combinations of side chains are impossible for the *targeted MWs*, the branch and bound algorithm in NP-StructurePredictor iteratively searched the best side chain candidates starting from extended position 1 to position *S* on the considered scaffold, and omitted impossible combinations in each iteration. Suppose we are searching all combinations of side chains on a scaffold with *S* atom-positions, and the algorithm searched a possible side chain combination when only positions from 1 to y (*y < S*) have been processed. The current selected combination can be skipped once the MW of current combination is larger than the *targeted MW.* Thus the algorithm can save execution time by only branching to appropriate combinations.

**An introduction of dynamic algorithm for structure elucidation**

The optimization problem of *CSCCP* has been defined in the main manuscript. The *CSCCP* can also be represented by a cost function defined as follows: in the CSCCP, $n$ denotes the number of substituted positions on a given seed scaffold, and $W_{0}$ is the *targeted MW*. A CSCCP cost function $C:\left\{ s|1\leq s\leq n,s\in N \right\}\times\left\{ w|1\leq w\leq W_{0},w\in N \right\}\times\{r|1\leq r\leq R,r\in N\}\to\left[ 0, 1 \right]$*,* is defined such that $C(s,w,r)$ stands for the highest $r^{th}$ value of $\prod_{i=1}^{s} p_{i{,x}_{i}}$ when only *s* out of *n* positions are extended by side chains and total molecular weight of the selected side chains is equal to *w* $(\sum_{i=1}^{s} m_{i,x_{i}}=w)$*,* where *w* is an integer number. The $x_{i}$*^th^* substituent at position $i$ of the given scaffold has a known probability $p_{i,x_{i}}\in[0, 1]$, representing its frequency of occurrence in nature, and a molecular weight $m_{i,x_{i}}$*,* which is a nonzero positive floating point number. If the original molecular weights $W_{0}$ and $m_{i,x_{i}}$ are floating point numbers, they are transformed into integers for the following analysis. Therefore, $C(s,w,r)$ corresponds to a sub-problem of the CSCCP when only *s* substituted positions are considered. The *r* highest values of $\prod_{i=1}^{s} p_{i{,x}_{i}},x_{i}\in\left\{ 1,2,\ldots{,K}_{i} \right\},\forall i\in\left\{ 1,2,\ldots,s \right\}$ are denoted by $C(s,w,1:r)$*,* where *“1:r”* denotes *“from 1 to r”.* The goal of the CSCCP is to find the R highest values $C(n,W_{0},1:R)$ *of* $\prod_{i=1}^{n} p_{i,x_{i}}$ satisfying $\sum_{i=1}^{n} m_{i,x_{i}}=W_{0},$where$“1:R”$ denotes *“from 1 to R”.* Thus, the problem of finding the potential structures in the *CSCCP* can be regarded as solving a mathematical procedure of the cost function$C\left( n,W_{0},1:R \right)$. To set the initial configurations of the cost function, in $C(0,w,r)$, for any values of *w* and *r*, $C(0,w,r)$ is 0, except for the case of $C\left( 0,0,1 \right),$ in which $C(0,0,1)$ is equal to 1.

Next, we utilized the dynamic programming strategy to iteratively compute the cost function based on the initial condition. To solve the entire *CSCCP* problem,$C(n,W_{0},1:R)$, we compute the sequence of the sub-problems, $C(s,w,1:R)$ for $s=1, 2, \ldots, n$, and $w=1,2,\ldots,W_{0}$. Given the values of $C\left( s-1, 1:w, 1 \right)$, we can calculate the value of $C(s, w, 1)$ using the equation below: $C\left( s, w, 1 \right)=\max_{x_{s}\in\{1,2,\ldots{,K}_{s}\}} \{p_{s,x_{s}}\times C\left. (s-1, w-m_{s,x_{s}},1) \right.\}$. According to the principle of DP, the optimal solutions of $C(s, w, 1)$ can be decided by the optimal solutions in the previous step, $C\left( s-1, 1:w, 1 \right).$ Finally, the highest *R* potential structures (selected side chains) in $C(s,w,1:R$*)*, with $s\in\{1,2,\ldots,n\}$ and $w\in\{0,1,\ldots{,W}_{0}\}$ can be evaluated by $C\left( s, w, 1:R \right)=\max_{{top R, x}_{s}\in\left\{ 1,2\ldots{,K}_{s} \right\},r\in\{1,2\ldots,R\}} \{p_{s,x_{s}}\times C\left. \left( s-1, w-m_{s,x_{s}},r \right) \right.\}$, where *K_s_* is the number of possible side chains at the *s^th^* substituted position on the given scaffold and $m_{s,x_{s}}$and $p_{s,x_{s}}$ are the molecular weight and probability of the ${x_{s}}^{th}$ side chain that can be linked on the *s^th^* substituted position.

Reference:

*1. Corporation -N. CUDA C Programming Guide. 5.5 ed2013.*

*2. Bemis GW, Murcko MA. The properties of known drugs. 1. Molecular frameworks. J Med Chem. 1996 Jul 19;39(15):2887-93.*
